# Cholesterol and Its Metabolites in Tumor Growth: Therapeutic Potential of Statins in Cancer Treatment

**DOI:** 10.3389/fendo.2018.00807

**Published:** 2019-01-21

**Authors:** Adele Chimento, Ivan Casaburi, Paola Avena, Francesca Trotta, Arianna De Luca, Vittoria Rago, Vincenzo Pezzi, Rosa Sirianni

**Affiliations:** Department of Pharmacy and Health and Nutritional Sciences, University of Calabria, Cosenza, Italy

**Keywords:** cholesterol, statins, cancer treatment, lipid raft, oxysterols, steroids, vitamin D, ERRalpha

## Abstract

Cholesterol is essential for cell function and viability. It is a component of the plasma membrane and lipid rafts and is a precursor for bile acids, steroid hormones, and Vitamin D. As a ligand for estrogen-related receptor alpha (ESRRA), cholesterol becomes a signaling molecule. Furthermore, cholesterol-derived oxysterols activate liver X receptors (LXRs) or estrogen receptors (ERs). Several studies performed in cancer cells reveal that cholesterol synthesis is enhanced compared to normal cells. Additionally, high serum cholesterol levels are associated with increased risk for many cancers, but thus far, clinical trials with 3-hydroxy-3-methylglutaryl coenzyme A (HMG-CoA) reductase inhibitors (statins) have had mixed results. Statins inhibit cholesterol synthesis within cells through the inhibition of HMG-CoA reductase, the rate-limiting enzyme in the mevalonate and cholesterol synthetic pathway. Many downstream products of mevalonate have a role in cell proliferation, since they are required for maintenance of membrane integrity; signaling, as some proteins to be active must undergo prenylation; protein synthesis, as isopentenyladenine is an essential substrate for the modification of certain tRNAs; and cell-cycle progression. In this review starting from recent acquired findings on the role that cholesterol and its metabolites fulfill in the contest of cancer cells, we discuss the results of studies focused to investigate the use of statins in order to prevent cancer growth and metastasis.

## Introduction

Cholesterol homeostasis is required for the normal growth of eukaryotic cells. Cholesterol is needed within cell membranes where it regulates membrane fluidity, signaling initiation, and cell adhesion to the extracellular matrix (1). In mammals, cholesterol is a precursor for bile acid and steroid hormone synthesis, cholesterol can be derived from food or synthesized *de novo* by specialized cells. The low-density lipoprotein (LDL) receptor (LDLR) is the primary pathway for removal of cholesterol from the circulation (2) and its activity is accurately controlled by intracellular cholesterol levels (3). The biosynthetic pathway of cholesterol is highly conserved, from yeast to humans. In the mitochondria, citrate, derived from the tricarboxylic acid (TCA) cycle, is converted to acetyl-coenzyme A (acetyl-CoA) beginning cholesterol synthesis. In the endoplasmic reticulum, acetyl-CoA is converted to lanosterol through a cascade of enzymatic reactions known as the mevalonate pathway. This series of reactions is regulated by a rate-limiting step catalyzed by 3-hydroxy-3-methylglutaryl-coenzyme A (HMG-CoA) reductase, which converts HMG-CoA to mevalonate. Downstream products of mevalonate, include cholesterol, ubiquinone, dolichol, and the isoprenoids geranylgeranyl pyrophosphate (GGPP) and farnesyl pyrophosphate (FPP) that bind to several small GTP-binding proteins such as Ras and Rho. Protein prenylation facilitates protein translocation from the cytosol to the membrane, promoting protein-protein and protein-membrane interactions and regulating protein function (4).

Several studies reported that cholesterol plays critical roles in the progression of numerous cancers (5), suggesting how cholesterol accumulation represents another general feature of tumors. HMG-CoA reductase and LDL receptor activities are increased in proliferating cancer cells, causing a rise in cholesterol content and consumption (6, 7). Cholesterol synthesis is under feedback control (8, 9), which is operated at the level of mevalonic acid production (10–12). When exogenous cholesterol is not available, the synthesis of mevalonate is increased in order to maintain levels of cholesterol and isoprenoids. When exogenous cholesterol levels are elevated a negative feedback mechanism inhibits the synthesis of mevalonic acid (10–12), this is particularly true for the liver (9), but in some species (13) for all tissues, blocking the overall *de novo* synthesis of endogenous cholesterol. In malignant cells this feedback mechanism is lost (10).

Statins are cholesterol-lowering drugs, which inhibit the rate-limiting step of conversion of HMG-CoA to mevalonate (14). Statins can be differentiated into two types, based on their solubility: hydrophilic (pravastatin, rosuvastatin) and lipophilic (simvastatin, lovastatin, fluvastatin, atorvastatin) statins. Several reports propose a promising role for statins in cancer treatment (15). Observational studies have tried to evaluate the effect of statins on patients with several cancer types such as prostate (16), colorectal (17, 18), renal cell carcinoma (19), breast (20), ovarian (21), and lymphoma (22). Results are variable, with some studies suggesting longer survival, and others reporting no benefit. Epidemiological evidences are also variable, depending on the cancer particular type as well as on the statin class used (23). Several data suggest that lipophilic statins may be preferable over the hydrophilic ones as anticancer agents (24). Statins anticancer properties could be explained through their pleiotropic effects such as lowering protein prenylation (17), reducing tumor cell proliferation and migration (20, 25), inhibiting Ras signaling (26) inducing apoptosis through inactivation of Akt and down-regulation of mammalian target of rapamycin (mTOR) (27).

Statins can interfere with cholesterol activities, which include:

- signal molecule on membrane rafts;
- substrate for steroids, oxysterols and Vitamin D3 synthesis;
- ligand for estrogen-related receptor alpha (ERRα).

In this review, we will discuss the results of studies focused on the use of statins to the purpose of interfering with cholesterol activities, in order to prevent cancer growth and metastasis.

## Cholesterol as Signal Molecule on Membrane Rafts

Cholesterol can act as a signaling molecule at the membrane rafts (28). It is well established that cholesterol-rich microdomains in the plasma membrane and lipid rafts constitute centers of organization for signal transduction and intracellular transport (29). Rafts are small functional areas of the plasma membrane, rich in sphingolipids and cholesterol. These regions are fluid but more ordered and tightly packed than the surrounding bilayer, because of saturated fatty acids. Rafts and related membrane microdomains, such as caveolae characterized by high caveolin-1 expression, have been proposed to play important roles in sorting of membrane molecules and in signal transduction in animal cells (30). Glycophosphatidylinositol-anchored (GPI-anchored) proteins; doubly acylated proteins, such as tyrosine kinases of the Src family, Gα subunits of heterotrimeric G proteins, endothelial nitric oxide synthase (eNOS), and cholesterol-linked and palmitate-anchored proteins are examples of molecules that can be found at membrane rafts (31).

Growth factors signaling are often deregulated in cancer cells. Insulin-like growth factor-I (IGF-I) through its receptor (IGF1R) is one of the most potent natural activators of the phosphatidylinositol 3-kinase (PI3K) and its downstream target Akt, which participate in cell survival pathway. The PI3K/Akt pathway is compartmentalized within plasma membrane raft domains (32). Activated PI3K/Akt control of cell proliferation, apoptosis and tumorogenesis, and aberrant activation of PI3K/Akt pathway contributes to the development and invasiveness of cancer cells (33, 34).

Other studies suggest that, within lipid rafts localize both epidermal growth factor receptor (EGFR) and Human Epidermal growth factor Receptor 2 (HER2) and their signaling events are dependent on cholesterol content of the lipid-rafts (35). Once again, the disruption of the lipid rafts, via depletion of circulating cholesterol levels, interferes with the receptor activation, inhibiting cell growth and development (35–37).

Metastatic events are characterized by cell adhesion decrease and cell migration promotion. Integrins and cell surface glycoproteins such as CD44, have a central role in adhesion mechanisms. CD44 is an adhesion molecule associated with lipid rafts and expressed in several cancers (38, 39). It has been demonstrated that ligand-binding ability of CD44 to hyaluronan, is governed by its cholesterol-dependent localization to cell membrane microdomains (40).

Several cancer cell types, including breast and prostate, have higher membrane cholesterol levels and are richer in membrane rafts (probably as a result of cholesterol accumulation) than their normal counterparts. For this reason, cholesterol-depleting agents are more effective in inducing apoptosis on these cancer cells than on normal cells (41, 42). Rafts/caveolae are rich in various signaling molecules and they have been associated with a number of functions, including cell survival, proliferation, and migration (28, 43).

It has been reported that apoptotic pathways, both extrinsic (death receptor pathway) and intrinsic (mitochondrial), are associated with lipid rafts because changes in cholesterol content within specific membrane regions regulate apoptotic signaling events (41, 44). Signalings from death receptors Fas receptor (FasR) and TNF-related apoptosis-inducing ligand (TRAIL) receptors 1 and 2 are strictly dependent on translocation into lipid rafts (44, 45). In fact, TRAIL- and Fas-mediated apoptosis is down-regulated by lowering membrane cholesterol in non-small cell lung carcinoma (46) and human Jurkat T leukemia cells (44).

Very recently, disruption of lipid rafts on breast cancer (BC) cell lines MDA-MB231 and MDA-MB468, using the cholesterol depleting agent methyl-β-cyclodextrin, resulted in reduced proliferation and migration, induction of apoptosis evidenced by cell shrinkage, membrane blebbing, nuclear condensation, and chromatin cleavage (47). It was already demonstrated for BC, that lipid rafts disruption causes decreased migration and invasion downregulating caveolin-1 along with urokinase-type plasminogen activator receptor (uPAR) and matrix metallopeptidase 9 (MMP-9) (48). In general, proteins identified in cancer lipid rafts include those involved in endocytosis, Src signaling, cytoskeletal remodeling, chaperones, extracellular matrix (ECM) remodeling (49).

### Statins and Lipid Rafts

Based on the importance of cholesterol at the raft/caveola, cholesterol depletion from the plasma membrane would disrupt intracellular signaling triggered by cell surface receptors (50). Statins lower cellular cholesterol content and thus are useful in the analysis of lipid-raft function. However, the effect on raft/caveola formation in cancer cells after statins treatment are not completely defined.

Menter et al. (51), evidenced that simvastatin inhibited the growth of several tumor cell lines with a time-dependent behavior. A significant reduction in cellular cholesterol level were observed after simvastatin treatment, starting at 24 h and up to 72 h. During this time frame, authors observed a reduction in cholesterol content at membrane rafts, caveolin-1 phosphorylation inhibition, disruption of caveolae and loss of membrane integrity. However, cholesterol depletion affected membrane signaling also in caveolin-negative cells (52). Specifically, using the prostate cancer (PCa) cells LNCaP, which do not express caveolin, simvastatin lowered raft cholesterol content, inhibited Akt signaling and induced apoptosis. In addition, using the same cells grown as xenografts, authors demonstrated that elevation of circulating cholesterol using a cholesterol enriched diet, promoted tumor growth and survival, as a consequence of activated Akt signaling via cholesterol-rich lipid rafts (52).

In another study, four head-and-neck squamous cell carcinomas (HNSCCs), four cervical carcinomas, five non small cell lung cancers (NSCLCs), four colon carcinomas, the epidermoid carcinoma cell line A431, and the breast adenocarcinoma MCF-7 were treated with lipophilic lovastatin, which inhibited EGF-induced EGFR autophosphorylation and its downstream signaling cascades (53).

Similarly, another hydrophobic statin, simvastatin caused in A431 cell line anoikis-like apoptosis, characterized by decreased raft levels, Bcl-xL down-regulation, caspase-3 activation, and Akt inactivation (41).

In PC-3 cells simvastatin treatment down-regulated IGF1R expression (54) and inhibited both basal and IGF-1-induced ERK and Akt activation (55). Raft modulating agents are more effective in cells containing a higher content of lipid rafts. In fact, breast (MCF-7, MDA-MB231) and prostate (PC-3, LNCaP) cancer cell lines were more sensitive to cholesterol depletion-induced cell death than normal breast and prostate cell lines (MCF-10A and PZ-HPV7, respectively) (41).

Garnett et al. examined the effects of MβCD and the hydrophilic paravastatin on cholesterol-rich rafts and caveolae and on gene transcription in MDA-MB231 and CaLu-1, lung adenocarcinoma. Both treatments caused a downregulation of genes involved in signal transduction, chemokine and anti-apoptotic pathways. Pravastatin increased expression of caveolin-1, but caveolae density was decreased, because of caveolin-1 inability to properly complex with cholesterol in an altered sterol environment. Similarly, MβCD caused an increase in caveolin-1 expression and reduced both rafts and caveolae, however, it had less effects on gene transcription. Indeed, signaling are more profoundly affected by statins than by the cholesterol-sequestering drug, indicating that not only cholesterol but also some intermediates of cholesterol synthesis downstream of mevalonate, play an important role in signaling pathways at the caveolae (56).

It has been reported that primary cells, HEL, SET-2, and UKE-1, derived from myeloproliferative neoplasms (MPNs) patients require mutated Janus kinase 2 (JAK2), responsible for increased growth signaling (57, 58). Simvastatin disrupts lipid raft and has a negative effect on mutated JAK2-dependent signaling. More specifically, in MPNs cells, simvastatin, lovastatin and atorvastatin inhibited mutated JAK2 localization to lipid rafts reducing cell viability, inducing apoptosis and inhibiting colony formation (59). Colony formation assay is considered a 3D cell culture assay where cells grow independently of a substrate (it is also known as anchorage-independent growth). This assay is particularly useful when studying long-term effects of drugs on anchorage-independent growth of cancer cells. Colony formation evaluates the ability of tumor cells, escaped from the primary tumor site, to exit the blood flow and initiate the post-intravasation phases of metastasis. CD44, as adhesion molecule, plays a central role in the progression of metastasis. The modulation of cholesterol either by statin or MβCD causes dissociation of CD44 from the lipid rafts (40) suggesting that membrane cholesterol may impact metastasis formation. Similar to the results on CD44 cholesterol depletion triggers the shedding of several molecules involved in cancer cell adhesion, including CD30 (60), L1-CAM (61) and collagen types XVII (62) and XXIII (63).

Activity of Ras-Related C3 Botulinum Toxin Substrate 1 (Rac1), a member of the Rho family of Small GTPase, is dependent upon its localization in membrane rafts and its activation is correlated with invasion and metastasis. Cholangiocarcinoma cells treated with simvastatin lose Rac1 rafts localization, because of decreased total cellular cholesterol and disruption of membrane rafts. In normal human cholangiocytes, simvastatin reduced cholesterol level, but did not affect Rac1 localization. In addition, simvastatin inhibited cell proliferation but, differently from cancer cells, it did not lead to apoptosis (64).

All these data unequivocally suggest that statins have a direct impact on membrane rafts, reducing signaling transduction and adhesion mechanisms in several cancer cell types, thus interfering with cell proliferation and metastasis.

## Cholesterol as Substrate for Steroids and Oxysterols Synthesis

Steroid hormones are synthesized from cholesterol in gonads, adrenal cells and placenta (65). The *de novo* synthesis of some steroid hormones occurs also in the nervous system (66, 67), in cardiac tissue (68) and other peripheral sites (69).

Based on their physiological function, steroid hormones are divided into five groups: mineralocorticoids, which act on the kidney to retain sodium; glucocorticoids, involved in the regulation of glucose metabolism; estrogens, which induce female secondary sexual traits; progestins, which are essential for reproduction; and androgens, which induce male secondary sexual characteristics. These classes of hormones contain the cyclopenta-phenanthrene nucleus and arise from reactions catalyzed by enzymes that belong mainly to the family of cytochrome P450 (CYP). They bind to specific steroid hormone receptors, which act as transcription factors. The active hormone/receptor complexes regulate transcription of distinct set of genes in a tissue-specific manner.

Oxysterols represent 27 carbon-atom molecules derived from cholesterol oxidization through enzymatic processes, or by-products of the cholesterol biosynthetic pathway. Considering the shorter biologic half-life when compared to cholesterol, oxysterols can be considered a way to route cholesterol for catabolism. Specific CYP, localized within the mitochondria or endoplasmic reticulum, are responsible for oxysterol synthesis (70, 71). Among them, the most abundant in human serum are 27, 24(S), 7α, and 4β hydroxycholesterol (HC). The 24(S)HC is synthesized by cholesterol hydroxylase encoded by CYP46A1 in neurons of the central nervous system (72). 7α and 27HC are synthesized in the liver by CYP7A1 and CYP27A1, and represent, respectively, the first intermediates of classic and acidic bile acid synthetic pathways (73). However, 27HC and its synthesizing enzyme CYP27A1 are found also in other cell types (74). Lastly, 4βHC is generated by CYP3A4, a hepatic drug metabolizing enzyme.

Oxysterols act as ligands of Liver X Receptors (LXR) α (NR1H3) and β (NR1H2) (75) to regulate transcription of specific genes. LXRα is expressed primarily in liver, intestine, adipose tissue and macrophages (76), and adrenal (77), whereas LXRβ is expressed in many cell types (78).

LXRα is also involved in the regulation of the adenosine triphosphate-binding cassette (ABC) proteins A1 and G1, cholesterol transporters involved in the flux of cholesterol from enterocytes and macrophages, respectively (79–82). LXRs also seem to have a role in the regulation of human cholesterol ester transfer protein (CETP), which translocates cholesterol esters between lipoproteins (83). Either steroid hormones or oxysterols activate proliferative and metastatic pathways in cancer cells.

### Steroid Dependent-Cancer Growth and Progression

It is well known that estrogens exert their biological effects interacting with two members of the nuclear receptor (NR) family, estrogen receptor α (ERα) or estrogen receptor β (ERβ) (84) and with a G-protein coupled receptor namely GPER (85). All three receptors can act at the cell membrane to activate signaling transduction pathways that ultimately regulate gene expression (rapid signaling). Additionally, ERα and ERβ can bind promoter regions of target genes, modulating their transcription (nuclear action). They directly bind DNA at estrogen response elements (EREs) located within promoters of estrogen-regulated target genes. Alternatively, they indirectly bind DNA through the interaction with transcription factors (TFs) that directly bind gene promoters. These TFs are stimulating protein 1 (Sp1) (86), activator protein (AP)-1 (87), nuclear factor-κB (NF-κB) (88). The oncogenic role of estrogens is well characterized in carcinomas of hormone-sensitive tissues including breast, prostate, endometrium and ovary, as well as in non-classical estrogen target tissues such as, adrenal, colon, and lung (89).

Genes involved in cell survival and proliferation and regulated by ERs, through direct or indirect binding to DNA, include myelocytomatosis viral oncogene homolog (MYC), cyclin D1 (CCND1), member RAS oncogene family 17 (RAB17), eukaryotic translation initiation factor 3 subunit A (EIF3A), and tumor protein D52-like 1 (TPD52L1) (90–92). Additionally, membrane ERs engage a functional crosstalk with growth factor receptors, including epidermal growth factor receptor (EGFR), insulin receptor (IR), insulin-like growth factor receptor (IGFR). Growth factor signaling can activate ERα in a ligand independent fashion through phosphorylation, and at the same time, estrogens can regulate IGF signaling (93). Treatment of ERα positive BC with selective estrogen receptor modulators (SERMs) such as tamoxifen, often leads to resistance. EGFR and/or IGFR are critical for the resistance to endocrine therapies (94). Additionally, transactivation of EGFR has been observed in MCF7 breast cancer cells via tamoxifen-dependent activation of GPER (95). The use of tamoxifen on patients with initial GPER-positive tumors increased GPER protein expression, and survival of these BC patients was markedly reduced (95).

Currently, a hot topic in the field of BC research is the definition of the role of androgens and the androgen receptor (AR), with studies revealing both tumor promotion and inhibition (96–98). Expression of AR is associated with favorable prognosis depending on the BC subtype and on whether ER is expressed or not (99, 100). Recently, dihydroxytestosterone (DHT) bound to AR has been shown to directly mediate epigenetic modifications of the chromatin, regulating expression of target genes (101). AR binds to ARE on target genes and, with the help of Lysine-specific demethylase 1A (LSD1), regulates histone modifications, demethylation by LSD1 at H3K4 of the E-cadherin promoter represses gene expression; similarly, LSD1 demethylation at H3K9 activates vimentin gene expression. Importantly, LSD1 is crucial for epithelial to mesenchimal transition (EMT) induction in several cancer cells (102, 103).

Prostate cancer relays on distinct proliferative pathways, including the PI3K and RAS/RAF pathways downstream of membrane AR activation; dysregulation of these pathways in both early and late stage prostate cancer was demonstrated through genomic profiling (104). In prostate cancer, androgens, testosterone (T), and DHT stimulate proliferation and inhibit apoptosis. Androgen ablation using anti-androgens such as bicalutamide favors cancer regression. This event is related to a lower rate of cell proliferation and to an increased rate of cell death (105). However, many patients do not respond to this therapy and die of recurrent androgen-independent prostate cancer (AIPC), characterized by a high metastatic rate. A crosstalk between androgen-sensitive PCa cells, androgen-independent PCa cells, and PCa-derived stromal cells has been very recently highlighted (106). Adrenal dehydroepiandrosterone (DHEA) is metabolized to DHT in androgen-independent PCa cells (AR negative cells, AR-) as well as in stromal cells. DHT is able to activate AR in androgen sensitive PCa cells (AR positive cells, AR+). Crosstalk among these cells may increase the migration and invasion potential of androgen independent PCa cells via EMT, evidenced by induction of N-cadherin, Snail and vimentin (106). GPER seems to have a role in tumor growth and progression of triple negative breast cancers (TNBC), tumors that lack expression for ERα, progesterone receptor (PR) and HER2 (107, 108). AR directly binds to GPER promoter and treatment with DHT decreases its transcription, possibly by competitively blocking the binding of positive regulators of GPER transcription (109). This reduced GPER expression following DHT treatment, is associated with increased tumor growth (110). Indeed, GPER role in TNBC is still controversial, with some studies indicating GPER involvement in increased tumor growth and worse overall survival (OS) (108) and some others a positive correlation between GPER and OS (111).

Estrogens exert carcinogenic effects on the prostatic epithelium. Combination of estradiol with low-doses of testosterone increased the incidence of prostate carcinomas in a rat model of PCa (112). Similar effects were observed in a mouse model of PCa. When ERα was knocked out in those mice, chronic treatment with testosterone combined with estradiol was unable to induce PCa. Additionally, mice had reduced PCa incidence when aromatase was knocked out. All together these data indicate that autocrine-produced estradiol working through ERα is determinant in PCa development (113). In agreement with animal studies, in the human prostatic epithelium ERα is overexpressed during the malignant transformation, supporting its role as an oncogene (114). On the contrary, ERβ is considered a tumor suppressor; in fact, its expression is decreased or lost in about 40% of PCa (115).

Inhibitory functions of GPER activation in prostate cancers has been demonstrated both *in vitro* and *in vivo* (116).

### Statins and Steroid Production

Statins, by decreasing cholesterol synthesis, will also affect the production of steroid hormones. Most steroid hormones are produced by the gonads and adrenal cortex from cholesterol, which is uptaken from the circulating LDL and HDL (117, 118). Since steroidogenesis requires an efficient intracellular pool of cholesterol, by reducing its synthesis, statin therapy could affect steroid production. Cortisol is a steroid hormone produced by the adrenal gland, is mainly released at times of stress, but in normal conditions, its production has a circadian rhythm (119). The effects of statin treatment on cortisol synthesis or cortisol levels were evaluated in several studies, that, however, did not demonstrate any significant effect of statins on cortisol levels (120, 121). An increase in plasma cortisol concentrations was highlighted by a recent meta-analysis study of data from seven randomized controlled trials with various statins (122). In general, the study demonstrated a higher impact of lipophilic statins (atorvastatin, lovastatin, and simvastatin), when compared to hydrophilic statins (pravastatin and rosuvastatin) (123). The increase in cortisol after statin treatment might explain the previously demonstrated anti-inflammatory effects of these drugs. The precise mechanism underling the rise in cortisol after statin use is not known. Studies evaluating the effect on hypothalamic-pituitary system or adrenal cortex itself should be performed to explain the mechanism activated by statins and responsible for the increase in cortisol levels. Based on the observation that only liphophilic statins, which have a greater non-hepatic distribution, affect cortisol levels, it can be speculated that statins have a direct effect on the adrenal gland. Enhancement of LDL-receptor expression, following the inhibition of adrenal HMG-CoA reductase, is responsible for increased cholesterol uptake allowing higher substrate availability for cortisol production. The effect of statins on the expression of steroidogenic enzymes involved in cortisol production is unknown.

A systematic review and meta-analysis of randomized controlled trials demonstrated that among 501 hypercholesterolemic men statins lowered testosterone; similarly, testosterone was reduced in a trial of 368 young women with polycystic ovary syndrome (PCOS) (124).

The direct effect of statins on HMG-CoA reductase in tumor cells is responsible for decreased substrate availability, lowering estrogens and androgens production that drive BC and PCa respectively. Recently, it has been found that statins and dehydroepiandrosterone sulfate (DHEAS) compete for the same transporter, SLCO2B1. Statin administration competitively reduces uptake of DHEAS and consequently tumor cell proliferation of PCa cell lines. The authors demonstrated that statin use at the time of androgen deprivation therapy initiation was associated with delayed tumor progression (125).

In physiological conditions, the prostate is not a steroidogenic site; but steroids, particularly testis-derived testosterone and DHT, regulate its function. In the context of a tumor, prostatic cells become capable of autonomous steroidogenesis (126). Evaluation of statin effects on the expression of steroidogenic enzymes in PC3 cells, demonstrated no effects on steroidogenic acute regulatory protein (StAR), cytochrome P450 family 11 subfamily A member 1 (CYP11A1), cytochrome P450 family 17 subfamily A member 1 (CYP17A1), hydroxy-delta-5-steroid dehydrogenase, 3 beta- and steroid delta-isomerase 2 (HSD3B2), steroid 5 alpha-reductase 2 (SRD5A2), and aldo-keto reductase family 1 member C2 (AKR1C2). Conversely, the expression of 17β-hydroxysteroid dehydrogenase type 5 (AKR1C3) was increased and 3β-hydroxysteroid dehydrogenase 1 (HSD3B1) was decreased. These changes in gene expression are responsible for the increase in DHT and T observed following simvastatin treatment (55).

All these studies suggest that steroidogenic tissues are potential sites for statins effect. On the adrenal gland there is no clear modulation of cortisol production, with only lipophilic statins having a direct effect on the adrenal and increasing cortisol synthesis. On the testis and PCOS statins reduce T production. In the context of tumors, there are discording data, reporting both decrease and increase in T production.

### Oxysterols Dependent-Cancer Growth and Progression

Epidemiological studies evidenced that high levels of dietary cholesterol would increase the risk of BC in postmenopausal women and the risk of cancers of the stomach, colon, rectum, pancreas, lung, kidney, bladder and non-Hodgkin lymphoma (127). This is probably due to increased oxysterol production which parallels hypercholesterolemia. Elevated concentrations of oxysterols have been associated with colon (128), lung (129), breast (130, 131), skin (132), prostate (133), and bile duct (134) cancers.

Among oxysterols, 27HC is synthesized by CYP27A1, which has a broad substrate specificity and is present in most tissues and not only in the liver. However, 27HC is not an efficient activator of human LXRs (75), instead has been identified to bind ERs (135). 27HC, binds the ERα on epithelial cells of the mammary gland and promotes BC growth (130, 131), while binding LXRα increases metastasis in the MMTV-PyMT mouse model of BC. Similarly, 25HC enhances cell proliferation of a breast cancer cell line via the activation of ERα target genes (136). Both 25HC and 27HC increased the transcription of ER target genes in long-term estrogen deprived BC cell lines, suggesting that these oxysterols replace estrogens and activate ER-mediated gene expression. This event can explain a mechanism involved in the development of resistance to aromatase inhibitors (137). Interestingly, BC patients treated with aromatase inhibitors had significantly increased plasma levels of 27HC and moderately increased levels of 25HC after 28 days of treatment (138), supporting a potential role of 25HC and 27HC level in patient outcome.

More recently, effects of 27HC have been studied in both androgen-responsive LNCaP (AR+) cells and androgen-irresponsive PC3 (AR-) prostate cancer cells. Both cell types increased proliferation in response to the oxysterol binding to ERβ (133).

Oxysterol-binding protein (OSBP) (139) and OSBP-related proteins (ORPs) were originally isolated because of their ability to bind oxysterols, and later cholesterol (140). They comprise a 12-member mammalian gene family, characterized by a conserved OSBP homology domain (OHD) that binds sterols and lipids, as well as the pleckstrin homology (PH) domain and two phenylalanines in an acidic tract (FFAT) motif that mediate interaction with organelle membranes. Upon binding to cholesterol, OSBP promotes ERK (extracellular signal regulated kinase) activity and hence cellular proliferation (140). Among them ORP4, also known as OSBP2, is expressed constitutively in brain, heart and testis, where is expressed as three variants, ORP4L, ORP4M, and ORP4S. Recently, cell growth regulatory activity has been evidenced for ORP4 (141). ORP4 binds sterols and phosphatidylinositol 4-phosphate (PI4P), and binding activity is required for ORP4 to promote cell proliferation and survival (141). Silencing of all ORP4 variants (ORP4L, ORP4M, ORP4S) in HEK293 and HeLa cells inhibited cell proliferation and promoted growth arrest without inducing cell death (141). ORP4L promoted proliferation of three different cervical carcinoma cell lines. (142). ORP4 has been identified as high-affinity cellular receptor for a group of natural products named ORPphilins that potently inhibit the growth of human cancer cell lines (143). Administration of 25HC, a high-affinity ligand for ORP4, suppressed ORPphilin activity.

### Statins and Oxysterols Production

A recent phase II clinical trial aimed to investigate effects of statins on BC growth related to a reduction in 27HC levels. Patients were treated with 80 mg/day of atorvastatin to investigate the impact of statin treatment on serum 27HC and on tumor-specific CYP27A1 expression. Atorvastatin exhibited an anti-proliferative effect evidenced by changes in Ki67 index, which did not significantly correlate with changes in either serum 27HC or changes in intratumoral CYP27A1 protein expression. Collectively these data indicate that the anti-proliferative responses to statin treatment do not depend on 27HC reduction (144).

However, 27HC can still affect proliferation of BC resistant to aromatase inhibitors (AI). It has been suggested that AI-resistant tumors can still proliferate in response to 27HC, which similarly to E2 activates ERs (131). In fact, despite lower estrogen levels, aromatase inhibitors resistant tumors have extensive ERα binding to target genes. This is due to ERα activation by 27HC synthesized consequently to stable up-regulation of the entire cholesterol biosynthetic pathways, including genes involved in 27HC biosynthesis. Statins, reducing cholesterol, reduce 27HC, and decrease ERα binding to DNA, abrogating cell invasion (145).

Statin treatments do not seem to have any beneficial effect on the rate of appearance of prostate cancer, but definitively has an effect on the incidence of advanced PCa (146–148). Moreover, in the PC3 prostate cancer cell line, statins prevent the cell migration potential therefore reducing the formation of metastatic prostate colonies; however, the mechanism relies on inhibition of prenylated proteins, not on inhibition of oxysterol formation (149).

These observations suggest that the reduction of oxysterol production by statins treatment could have effect on specific tumors preventing cell migration and invasion.

## Cholesterol as Substrate for Vitamin D Synthesis

Cholesterol is the precursor molecule for vitamin D. There are two major isoforms of vitamin D: vitamin D_2_ (ergocalciferol), and vitamin D_3_ (cholecalciferol) (150, 151). UVB radiations are needed to synthesize Vitamin D_2_ from ergosterol in plants, yeasts, and fungi and can be ingested from a diet containing food products of plant origin. In humans, vitamin D_3_ is synthesized from 7-dehydrocholesterol by UVB radiation in the epidermis of skin and can also be derived from the diet containing food products of animal origin.

Vitamin D is a prohormone that undergoes two-step metabolism in the liver by CYP27A1 to produce the 25(OH)D (calcidiol) and in the kidney by CYP27B1 to produce the biologically active metabolite 1α,25(OH)2D3 (calcitriol) which binds to the vitamin D receptor (VDR) regulating expression of diverse genes (152). CYP27B1 is also expressed in multiple extra-renal sites, including cancer cells (153). Thus, calcitriol can function in an endocrine (systemic) or autocrine manner when it is locally synthesized. Serum level of 30 to 50 ng/mL is normal for healthy people. Vitamin D deficiency and insufficiency is defined as serum 25-hydroxyvitamin D [25(OH)D] levels <20 and 21 to 29 ng/mL, respectively.

Recent studies have revealed that vitamin D can also be metabolized and activated through a CYP11A1-driven non-canonical metabolic pathway (154). The products of CYP11A1, such as 20(OH)D and its hydroxy-metabolites, produce differentiation, have anti-proliferative and anti-inflammatory effects in skin cells comparable or superior to calcitriol (155). Low vitamin D levels increase cancer risk, as evidenced by epidemiological, preclinical and cellular studies (154, 156). In particular, many *in vitro* studies, performed in several malignant cell lines, showed that the anti-cancer activity of this molecule is related to the inhibition of proliferation and angiogenesis and induction of apoptosis (157).

Epidemiological studies showed that serum levels of 25(OH)D adversely correlate with prostate cancer risk (158). In men living at high latitude, as in Scandinavia, 25(OH)D blood serum levels are below 16 ng/mL and the incidence of prostate cancer is high (159). It was observed that 1α,25(OH)2D3 inhibited proliferation and stimulated apoptosis of VDR-positive prostate cancer cells and, interestingly had an anti-inflammatory effect toward this subtype of prostate tumors (160).

In BC cells 1α,25(OH)2D3 caused cell cycle arrest, by interfering with cyclin-dependent kinases activity (161). Additionally, apoptosis can be activated by reducing bcl-2 and up-regulating p53 levels (162). Cell proliferation can be inhibited by 1α,25(OH)2D3, interfering with ER function. Specifically, 1α,25(OH)2D3 and its analogs down-regulate the expression of ERα, which in turn reduced estrogen-dependent activation of mitogenic signal (163). Another action of 1α,25(OH)2D3 against breast cancer cells is the down-regulation of aromatase expression (164).

Proliferation of colon cancer cells was inhibited by 1α,25(OH)2D3 and its analogs, that caused a cell cycle arrest at the G0/G1 phase. This was consequent to enhanced expression of p21 and p27, two cyclin-dependent kinase inhibitors, and to reduced expression of cyclin D1 and cyclin E (165). In addition, following colon cancer cells treatment with 1α,25(OH)2D3, genes with a pro-apoptotic function were increased, while those anti-apoptotic were downregulated (166). It was observed that in differentiated human colon tumors CYP27B1 expression is enhanced compared to untransformed colon mucosa. It was observed a parallel increase of VDR and CYP27B1 mRNA during early tumor progression. This suggests that 1α,25(OH)2D3 synthesized in colonocytes and bound to its receptor could exert its anti-mitotic function in both an autocrine and a paracrine fashion to prevent intestinal tumor formation and progression during early phases of colon tumorigenesis. In fact, in high-grade undifferentiated tumors, expression of CYP27B1 is decreased (167). However, other reports did not find a rise in CYP27B1 expression in tumors, possibly because there was not a distinction for the biological grade of the tumor (168). With a very similar pattern of expression, when compared with normal colon mucosa, VDR expression is increased in early stages of tumorigenesis, but declines in late-stage tumors (169–171). Alternatively, 1α,25(OH)2D3 produced during early phases of transformation could interact with other receptors such as thyroid receptors (TR) (172) and induce cell proliferation. In fact, the expression of TRβ1 was found associated with polypoid growth and with higher histological grade and advanced stage (173). To confirm a protective role for 1α,25(OH)2D3, CYP24A1, the degrading enzyme, has enhanced expression in the majority of colon adenocarcinomas, keeping low 1α,25(OH)2D3 levels (174). Adrenocortical cancers express VDR, its activation by slightly supra-physiological concentrations of 1α,25(OH)2D3 has a moderate anti-proliferative effect, that is related to cell cycle arrest, promoting accumulation of cells in G1 phase, without inducing apoptosis. Additionally, VDR activation decreases cortisol, aldosterone, and DHEA-S production (175).

A very recent study demonstrated no impact of statin therapy on plasma vitamin D levels (176). However, a meta-analysis report indicated opposite data, with statins causing an increase in vitamin D serum levels (177), this effect was observed with lovastatin (178), simvastatin (179), atorvastatin (180), and especially rosuvastatin (181, 182). If further confirmed, these data might help explaining the anti-neoplastic effect exerted by statins on colon cancer (183–185). Currently, there are no studies investigating the effects of statins on intratumor vitamin D synthesis despite vitamin D can act in an autocrine manner to regulate cancer growth. For these reasons further studies on specific tumor are necessary to establish a direct effect of vitamin D on cell tumor proliferation and consequently if statins could induce anti-tumoral effects modulating intra-tumoral vitamin D levels.

## Cholesterol as Estrogen-Related Receptor Alpha (ERRα) Ligand

The Estrogen-related receptor (ERR) family is known to comprise three members [ERRα (NR3B1), ERRβ (NR3B2), and ERRγ (NR3B3)] (186). ERRα is ubiquitously expressed in adult tissues; ERRβ is detected at low levels in the liver, skeletal muscle, stomach, heart, and kidney; ERRγ is widely expressed and can be detected in brain, lung, bon marrow, adrenal and thyroid glands, trachea and spinal cord. The ERRs, like most NRs, are organized in functional domains for ligand (LBD) and DNA binding (DBD), in addition to an activation function 1 (AF-1) involved in cofactors binding. ERα and ERRα LBD share only 37% amino acid homology, indicating low affinity for common ligands, and in fact estradiol fails in activating ERRα (186). While ERRβ and ERRγ are still orphan receptors since their natural ligands have not been identified, ERRα is an adopted orphan receptor, for which ligand has been identified to be cholesterol (187). This finding implies cholesterol involvement in mitochondrial metabolism and biogenesis. In fact, ERRα regulates the expression of a broad range of genes driving mitochondrial biogenesis, the tricarboxylic acid (TCA) cycle, and substrate oxidation.

ERR monomers preferentially recognize the consensus site referred to as the ERR-response element (ERRE). ERRα and ERα share 68% amino acid identity in the DBD, indicating that the two receptors can potentially regulate common genes. Indeed ERR dimers can bind to the ERE, and ERα dimers can also recognize ERRE sites (188). ERα and the ERRs compete for common coactivators such as steroid receptor coactivator (SRC) proteins in transfected cells (189). In addition, another coactivator, the small heterodimer partner (SHP), a coregulator of ER, interacts with all members of the ERR family inhibiting their transcriptional activity. Thus, ERRs and ERs have the potential to differentially regulate common target genes.

ERRα transcriptional activity in normal cells has important roles in cellular metabolism, this is particularly relevant in rapidly dividing cells such as tumor cells. Cholesterol interaction with ERRα (187) allows recruitment of coactivators PGC1α/β and increases ERRα transcriptional activity. ERRα interaction with PGC1α favors osteoclastogenesis (190) and bone reabsorption in osteoclasts, myogenesis (191) and decreases muscle toxicity in myocytes (187). Differently from other nuclear receptors, ERRα is constitutively active because cholesterol is ubiquitous, meaning that it does not require any spike in ligand concentration, as is the case for steroid hormone receptors. ERRα antagonists have been found to induce cancer cell death (192, 193), inhibit tumor growth (194) and improve insulin sensitivity and glucose tolerance (195).

The use of statins or drugs targeting the SREBP metabolic pathways could be a promising option to counteract ERRα-dependent metabolic rearrangement. Identification of cholesterol as ERRα ligand is relatively new, so far no studies have investigated statins effects on ERRα activity in tumor cells. However, the discovery of cholesterol as ERRα ligand has elucidated the mechanism behind statin-induced muscle toxicity (187).

## Conclusions

Statins are widely used drugs for their ability to lower cholesterol levels in hypercholesterolemic patients. Their mechanism of action consists in the inhibition of HMG-CoA reductase, the main enzyme involved in cholesterol biosynthesis.

Aim of this review was to discuss the results of studies focused on the use of statins to the purpose of interfering with cholesterol activities, in order to prevent cancer growth and metastasis.

**Figure 1:**
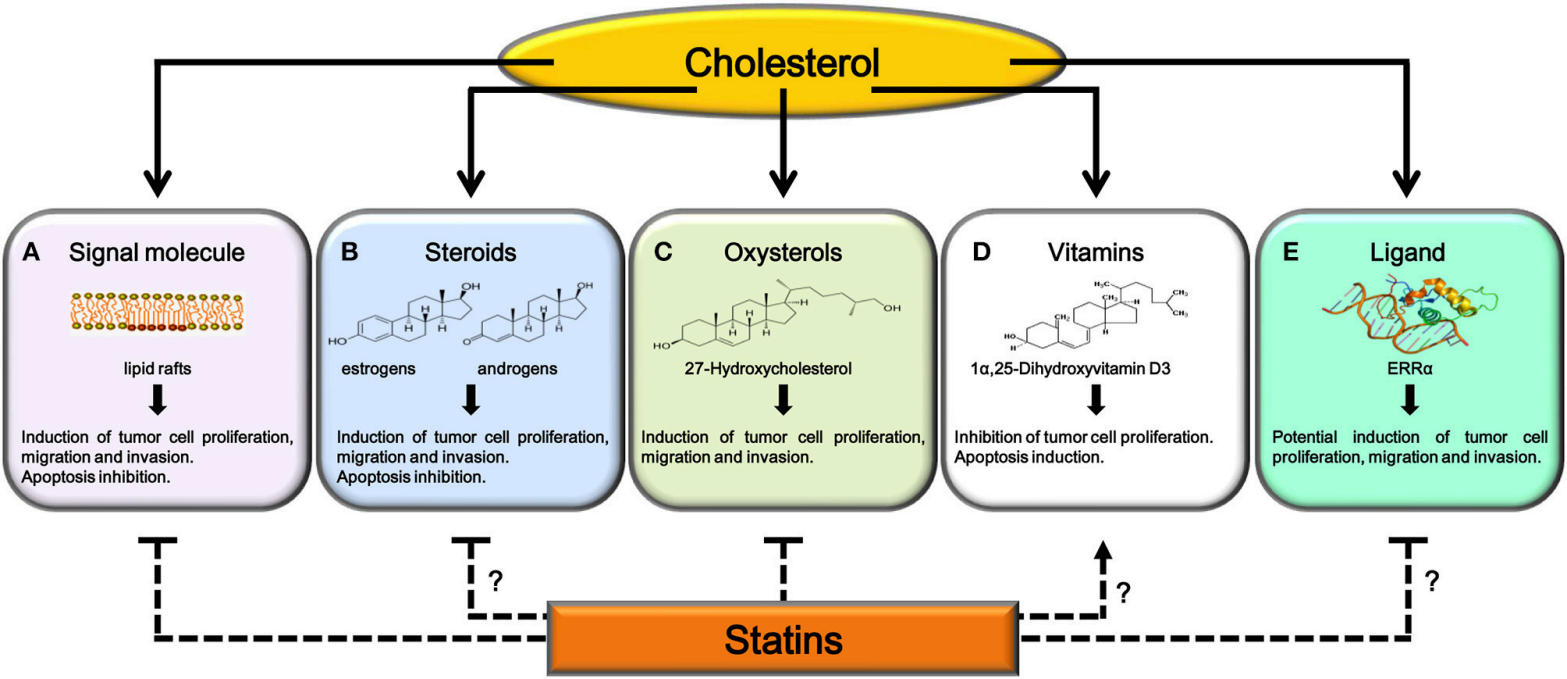
Potential mechanisms explaining antitumoral effects of statins. Cholesterol, after its utilization as signaling molecule **(A)**, as substrate for steroids **(B)**, oxysterols **(C)**, and 1α,25(OH)2D3 synthesis **(D)** or as ligand for ERRα **(E)**, regulates tumor growth and progression. Statins, inhibiting cholesterol synthesis, interfere with **(A)** and **(C)**. Further studies are needed to confirm statins ability to: reduce production of steroid hormones involved in cancer progression **(B)**, increase serum or intratumor 1α,25(OH)2D3 levels **(D)** or interfere with ERRα activity **(E)**.

Cholesterol plays an important function as part of membrane rafts where is involved in the modulation of signaling transduction related to cell proliferation and migration (Figure 1A). Data discussed herein unequivocally suggest that statins have a direct impact on the function of membrane rafts, inhibiting, in tumor cells, pathways regulating growth, and metastasis. Cholesterol represents a precursor for estrogens and androgens, hormones involved in modulating cell proliferation, migration, invasion and apoptosis in different cancers (Figure 1B). Even though steroidogenic tissues are potential sites for statins effects, there are discording data on a direct role for statins in decreasing steroid production in hormone-dependent cancers. Furthermore, using cholesterol as substrate for specific metabolizing enzymes it is also possible to produce oxysterols, such as 27HC, which has been shown to act as an endogenous selective estrogen receptor modulator able to increase cancer growth and metastasis (Figure 1C). Data discussed in this review suggest that the reduction of oxysterol production caused by statins could have a strong effect on specific tumors (i.e., breast cancer) preventing cell migration and invasion.

Cholesterol is also precursor of vitamin 1α,25(OH)2D3, which is involved in modulating VDR-responsive genes, encoding for proteins involved in anti-proliferative signaling (Figure 1D). However, the analysis of data published in this field suggest that further studies are necessary to establish a direct effect (anti-proliferative or proliferative) of vitamin D on different cell tumors and consequently if statins could induce specific effects modulating intra-tumoral vitamin D levels.

In conclusion, while the anti-tumor effects produced by statins on cholesterol-mediated transduction mechanisms at the membrane raft or on oxysterols synthesis, appear to be a promising therapeutic strategy, further studies are needed to determine if cholesterol depletion is a valid strategy to limit the effects of steroid hormones on endocrine-dependent tumors. The ability of statins to increase 1α,25(OH)2D3 serum levels need to be confirmed, in order to define another antitumor mechanism for these drugs. Recently, the discovery of cholesterol as ERRα ligand has elucidated the mechanism behind statin-induced muscle toxicity; however, no studies have investigated statins effects on ERRα activity in tumor cells (Figure 1E). This last aspect has opened a new field of investigation, in fact, strategies aimed to reduce cholesterol levels, such as the use of statins or drugs targeting SREBP metabolic pathways, could be a promising option to counteract metabolic rewiring in cancer cells where ERRα plays a pivotal role.

Preclinical studies support the potential use of statins as anticancer agents. Epidemiological studies indicate that statin use is associated with a reduction in the incidence of some tumor types. The few clinical trials of statins as monotherapy do not provide convincing results; however, in combination therapy with other agents, statins have shown more promising data. Conclusion of clinical trials not yet completed and publication of data from closed trials will provide a wider picture on the effectiveness of this class of drugs as anticancer therapy.

## Author Contributions

AC, IC: literature search and drafting of the article. FT, PA, AD, VR: drafting of the article. VP, RS: critical revision of the article and final approval.

### Conflict of Interest Statement

The authors declare that the research was conducted in the absence of any commercial or financial relationships that could be construed as a potential conflict of interest.
